# Relationship between obesity and early failure of total knee prostheses

**DOI:** 10.1186/1471-2474-10-29

**Published:** 2009-03-05

**Authors:** Barbara Bordini, Susanna Stea, Sara Cremonini, Marco Viceconti, Rossana De Palma, Aldo Toni

**Affiliations:** 1 Laboratorio Tecnologia Medica, Istituto Ortopedico Rizzoli, Via di Barbiano 1/10, Bologna, Italy; 2 Agenzia Sanitaria Regionale Emilia-Romagna, Viale Aldo Moro 21 – Bologna, Italy; 3 I divisione di Ortopedia e Traumatologia, Istituto Ortopedico Rizzoli, Via Pupilli 1, Bologna, Italy

## Abstract

**Background:**

Obesity is a risk factor for knee arthritis. Total knee arthroplasty is the definitive surgical treatment of this disease. Therefore, a high percentage of subjects treated are overweight. Since 2000 in the Emilia-Romagna Region the Register of Orthopedic Prosthetic Implantology, RIPO, has recorded data of all the primary and revision operations performed on the knee; height and weight of patients at the time of surgery have also been recorded.

**Methods:**

To understand how overweight and obesity affect the outcome of knee arthroplasty, a population of subjects treated with cemented total knee arthroplasty between 2000 and 2005 was studied. 9735 knee prostheses were implanted in 8892 patients; 18.9% of the patients were normal weight, 48.2% were overweight (25 < Body Mass Index <= 30), 31.1% were obese (30 < BMI <= 40), and 1.8% were morbidly obese (BMI > 40). Mean and range of follow-up were respectively 3.1 and 1.5–6 yrs. Implant failure was defined as the exchange of at least one component for whatever reason.

**Results:**

In normal weight patients there were 36 failures out of 1840 implants (1.96%), in overweight patients there were 87 out of 4692 (1.85%), in obese 59 out of 3031 (1.94%), and in morbidly obese there were 4 out of 172 (2.3%). The mean time to failure for each class was 1.57, 1.48, 1.60, 1.77 yrs. Cox regression analyses showed that the risk of implant failure was not influenced by BMI, absolute body weight, or sex. Conversely, an increased failure risk was observed in mobile meniscus prostheses in comparison with those with a fixed meniscus (Rate Ratio 1.88); an increased failure risk was also related to age (Rate Ratio 1.05 per year). These results were also confirmed when considering septic loosening as the end-point. There were no differences in the rate of perioperative complications and death in the 4 classes of BMI.

**Conclusion:**

In conclusion, cemented knee prostheses, implanted in patients with arthritis do not have significantly different rates of survival or perioperative complications in obese subjects compared with normal weight subjects, at least up to 5 years after surgery. The conclusion also applies to subjects affected by morbid obesity, altough this findings should be regarded with caution due to the small sample examined.

## Background

The prevalence of obesity in industrialized and emerging countries is reaching epidemic proportions. The growth of the population with an unhealthily high body weight is particularly relevant in the USA where 71% of the inhabitants over 60 years old are overweight or obese [1], although it has reached warning levels in many European countries [2-6] and Australia [7].

In Italy obesity is a public health problem: 39% of women and 50% of men over 65 of age are overweight and 15% and 14% respectively are obese. In the Emilia Romagna Region, in the North of the Country where most of the population considered in this study live, in the same age range, 42% of women and 55% of men are overweight and 13% and 14% are obese [8].

Obesity is related to several cardiovascular, metabolic, and osteoarticular diseases, especially arthritis of the knee and hip, and also other joints bearing smaller loads, thus suggesting both a mechanical and biochemical role in articular degeneration [9,10]. Treatment of arthritis is initially conservative using drugs and physiotherapy, but often, as the disease progresses, total joint arthroplasty becomes necessary. Therefore, many obese subjects can be found among those treated by total joint arthroplasty and the correlation between BMI (Body Mass Index) and osteoarthritis is now clear. Oliveria demonstrated that the risk of symptomatic osteoarthritis of the knee increased dramatically with increasing BMI (3.8 for BMI up to 30 and 9.3 for BMI > 30) [11]. Similarly, each unit in age-adjusted BMI has been shown to be associated with a 4% increase in incidence of osteoarthritis [12].

Based on these premises, the practice of knee joint arthroplasty is becoming more widespread worldwide [13-17] and its efficacy is undisputed [18].

Besides clinical experience, all implant Registers starting from 1975, following the example of Sweden, have confirmed the excellent results of this surgical procedure. In Italy RIPO (Register of the Orthopedic Prosthetic Implantology) began in 2000 in Emilia Romagna, a region counting 4.500.000 inhabitants; it captures 95% of the operations either primary and revision performed in the 61 public and private health structures of the Region [19]. Revisions undertaken in other regions of Italy are included, because the economic compensation among administrative services of Italian regions allows RIPO not to lose these patients to follow-up. Even if good results are generally obtained with total knee arthoplasy (TKA), some authors have raised doubts about its effectiveness in obese subjects, so much so that recently the East Suffolk Health trust prioritized non-obese (BMI < 30) in the selection of patients for lower limb joint replacement surgery [20].

The scientific community does not fully agree with this choice since there is no clear and definitive evidence of the effectiveness of the operation in obese patients. The literature, in fact, mainly shows the data of limited series, which do not provide a unanimity of views, both with regards to the different endpoints used in the definition of success, and the different stratifications performed in the patients.

The purpose of the present study was therefore to try to fill this gap and examine the relationship between body mass index and survivorship of knee prostheses on a register-based data-set.

## Methods

The data collected by RIPO in the period 2000–2005 were analyzed so as to have at least an 18-month follow-up. The study was performed on patients treated by fully cemented total knee prosthesis due to primary arthritis. This method of prosthesis fixation was used in 88% of the implants recorded in RIPO. A total of 11,529 knees were eligible for the study. Of these knees 1,794 (15.6%) were excluded due to missing information about weight or height, thus leaving 9,735 knees in 8,892 patients for evaluation. The outcomes of interest were compared between obese and non-obese patients. We used BMI to define obesity; according to the guidelines set by the World Health Organization BMI > 25 and < 30 Kg/m^2 ^was defined as overweight, BMI >= 30 kg/m^2 ^as obese, and BMI >= 40 as morbidly obese.

The distribution of the variables considered in the subsequent analyses (gender, age, type of insert) is shown in Table 1. They are not statistically different comparing the cases excluded with those included in the study. In the group of included patients, 567 died in the follow up period, 118 of them were normal weight, 275 overweight, 163 obese and 11 morbidly obese. The incidence is respectively 6.4%, 5.8%, 5.4%, 6.4%, not significantly different (Fisher exact test). In all cases the prosthesis was still in place at the time of death.

**Table 1:** Characteristics of the examined population.

EXAMINED	Men	Women	Total number (%)
Number of patients	2102	6790	8892
N. of prostheses	2281	7454	9735
Mean age at surgery (C.I 95%)	71.4 (71.1–71.7)	72.1 (71.9–72.2)	71.9 (71.8–72.1)
BMI			
Normal (BMI <= 25)	426	1414	1840 (18.9%)
Overweight (25 < BMI <= 30)	1252	3440	4692 (48.2%)
Obese (30 < BMI <= 40)	580	2451	3031 (31.1%)
Morbidly obese (BMI > 40)	23	149	172 (1.8%)
Type of insert			
Fixed	1612	5312	6924 (71.1%)
Mobile	669	2142	2811 (28.9%)

The most commonly used totally cemented knee prostheses in this study are presented in Table 2. They are grouped according to the type of insert, as this variable, in RIPO data, has been shown to influence the survival of the prosthesis [21].

**Table 2:** Types of cemented total knee prostheses implanted in the patients. Number of implants and failures are reported for each of them

FIXED INSERT		MOBILE INSERT	
n	Type	n	Type
1889/27	NEXGEN – LPS – Zimmer	484/15	PFC – RP – PS – De Puy Johnson & Johnson
1739/27	PROFIX – CONFORMING – Smith & Nephew	334/10	GENIUS TRICCC – Dedienne Sante
379/2	NEXGEN – LPS – FLEX FISSO – Zimmer	317/13	T.A.C.K. – Link
243/5	ADVANCE Medial Pivot – Wright	221/1	GEMINI MK II – Link
235/6	OPTETRAK – P.S. – Exactek	201/2	NEXGEN – LPS – FLEX MOBILE – Zimmer
215/2	913 – PS – Cremascoli Wright	175/8	ROTAGLIDE – Corin Medical
184/4	PFC – PS – De Puy Johnson & Johnson	154/7	LCS – UNIVERSAL – RP – De Puy J & J
171/11	INTERAX – Diamond Condilar – Stryker Howmedica	124/2	HLS – EVOLUTION ROTATOIRE – Tornier
154/1	SCORPIO – P.S. – Osteonics	801/19	Other (less than 100 each)
134/1	PROFIX – PLUS CONFORMING – Smith & Nephew		
121/4	DURACON II – LIPPED – Stryker Howmedica		
116/0	AGC V2 HPPS TOTAL KNEE – Biomet Merck		
111/0	SCORPIO – NRG – PS – Osteonics		
1233/19	Other (less than 100 each)		

The primary outcome in the analysis was revision of at least one component. The analysis was repeated using three different endpoints: revision of at least one component for whatever reason; revision of at least one component for sepsis; the exchange of poly liner for whatever reason. The insertion of a patellar button in a second stage was not considered as failure of the prosthesis according to the policy of the Register

The secondary outcome was the incidence of main complications during hospitalization. Complications were recorded by the surgeon at time of discharge from the hospital and transmitted to the register together with all the other information about the patient and prosthesis.

### Statistics

Cox proportional hazards regression was applied to estimate the effects of the different covariates on the failure rate. The hazard ratios are presented with ninety five percent confidence intervals. The end-point was revision of at least one component. Variables included gender, age, type of insert and BMI or weight. The Cox multiple regression model test enables verification of the influence of one variable on equal terms with the others [22].

## Results

The first primary end-point tested was the revision of at least one component for whatever reason. The result of multiple regression model is summarized in Table 3.

**Table 3:** Results of multiple regression model of knee prosthesis revision (whatever reason)

	Failures/Implants	Unadjusted (95% CI)	Adjusted for all variables* (95% CI)	P (adjusted model)
Gender				p = 0.223
Female	135/7454	1 (referent)	1(referent)	
Male	51/2281	1.29 (0.93–1.78)	1.22 (0.88–1.69)	
				
Insert				p = 0.0001
Fixed	109/6924	1 (referent)	1 (referent)	
Mobile	77/2811	1.85 (1.38–2.48)	1.88 (1.40–2.52)	
				
Age at surgery (for year)	186/9735	0.95 (0.94–0.97)	0.95 (0.94–0.97)	p = 0.0001
				
Body Mass Index				p = 0.965
Normal (BMI <= 25)	36/1840	1 (referent)	1 (referent)	
Overweight (25 < BMI <= 30)	87/4692	0.97 (0.66–1.43)	0.92 (0.62–1.35)	p = 0.661
Obese (30 < BMI <= 40)	59/3031	1.04 (0.69–1.57)	0.91 (0.597–1.382)	p = 0.654
Morbidly obese (BMI > 40)	4/172	1.29 (0.46–3.6)	1.02 (0.36–2.90)	p = 0.967

* Gender, type of insert, age at surgery, body mass index

In the analysis the total number of valid observations was 9,735 of which 9,549 were not removed and 186 were revised.

The outcome was not statistically significant affected by gender or by BMI class, but was affected by age and type of insert.

Then the analysis was repeated using the second primary end-point, which was septic loosening. In the analysis the total number of valid observations was 9735 of which 9677 were not removed and 58 were revised due to infection (Table 4).

**Table 4:** Results of multiple regression model of knee prosthesis revision due to infection.

	Failures/Implants	Unadjusted (95% CI)	Adjusted for all variables* (95% CI)	P (adjusted model)
Gender				p = 0.228
Female	40/7454	1 (referent)	1(referent)	
Male	18/2281	1.53 (0.87–2.66)	1.41 (0.81–2.48)	
				
Insert				p = 0.002
Fixed	31/6924	1 (referent)	1 (referent)	
Mobile	27/2811	2.26 (1.35–3.78)	2.28 (1.36–3.82)	
				
Age at surgery (for year)	58/9735	0.95 (0.92–0.99)	0.95 0.92–0.99)	p = 0.008
				
Body mass index				p = 0.851
Normal (BMI <= 25)	10/1840	1 (referent)	1 (referent)	
Overweight (25 < BMI <= 30)	31/4692	1.24 (0.61–2.53)	1.17 (0.57–2.38)	p = 0.67
Obese (30 < BMI <= 40)	16/3031	1.01 (0.46–2.22)	0.89 (0.40–1.98)	p = 0.78
Morbid obese (BMI > 40)	1/172	1.15 (0.15–8.99)	0.94 (0.12–7.49)	p = 0.96

* Gender, type of insert, age at surgery, body mass index

Both the first and the second analyses were repeated by repleacing some variable (weight instead of BMI, BMI as continuous variable), and adding the variable 'surgeon's experience' (surgeons having performed more than 180 knee arthroplasties in the period were considered experts). The results were unchanged even if the endpoint was limited to the exchange of the polyethylene insert (data not shown).

The results of the secondary outcome, i.e. the rate of complications during hospitalization are shown in Table 5. The rate was not statistically significant different in the 4 classes of BMI. Hospitalization duration was not statistically significant different in the 4 classes, as well, ranging between 11.7 and 12.5 days.

**Table 5:** Rate of intraoperative, local postoperative, and general complications and deaths according to class of Body Mass Index (BMI) of the patients treated by total knee arthroplasty.

	n. of trated patients	Patients with intraoperative complications (% of the total)	Patients with postoperative complications (% of the total)	Patients with general postoperative complications (% of the total)	Deaths in hospital
BMI <= 25	1840	9 (0.49) 5 bone fracture, 3 tendon rupture, 1 anest. complic	26 (1.4) 16 hematoma, 4 nerve injury 0 deep thrombosis 6 other	55 (3.0) 3 pulmonary embolism, 5 minor cardiac 14 acute anemia 5 urinary 28 other	2 (0.1)
25 < BMI <= 30	4692	13 (0.27) 4 bone fracture, 6 tendon/ligam rupture, 2 hemorrage 1 anest. complic	65 (1.4) 37 hematoma, 4 nerve injury 7 deep thrombosis 22 other	143 (3.0) 11 pulmonary embolism, 7 cardiac infarction 4 minor cardiac 32 acute anemia 17 urinary 72 other	5 (0.1)
30 < BMI <= 40	3031	7 (0.23) 3 bone fracture, 2 tendon/ligam rupture, 1 anest. complic	33 (1.1) 15 hematoma, 2 nerve injury 6 deep thrombosis 4 superficial infections 3 other	98 (3.2) 3 pulmonary embolism, 1 cardiac infarction 7 minor cardiac 17 acute anemia 15 urinary 55 other	1 (0.03)
BMI > 40	172	0 -	3 (1.7) 2 hematoma, 1 other	6 (3.5) 1 cardiac infarction 5 other	1 (0.58)
Total	9735	29 (0.30)	127 (1.3)	302 (3.1)	9 (0.1)

Rate of complications and deaths is not significantly different in the 4 classes of BMI. Fisher's exact Test > 0.05.

## Discussion

There is great interest in excess bodyweight in TKA, because obesity is closely related to knee arthritis and, as a consequence, because a high percentage of patients treated with TKA are overweight or obese.

The purpose of the present paper was to test, based on the data collected by the RIPO, the influence of obesity on outcomes of TKA. Only cemented prostheses implanted in patients with arthritis were considered, in order to reduce potential biases. Cementless or partially cemented knee prostheses represent, in fact, only 12% of all registered implants, and pathologies other than arthrosis only 11%.

The primary end-point selected, as applies to the analyses performed on the register data, was the removal of even only one prosthetic component either for septic or aseptic loosening.

The secondary end-point was the rate of complications and deaths observed during hospitalization.

The results obtained show that BMI does not influence the survival of the implant at a 5-year maximum follow-up due to either aseptic or septic loosening. No influence was observed for the rate of complications or deaths observed during hospitalization; length of hospitalization was comparable in the 4 classes.

BMI did not increase the risk of failure even in the few cases where it was extremely high, in patients with morbid obesity.

Furthermore, even absolute body weight does not appear to be a risk factor for implant failure. Differentiation between weight and obesity is important as one is an absolute measure and the other a relative one. A patient weighting 80 Kg is classified as normal if 180 cm high and obese if 160 cm high. The stress on the prosthesis is unmodified, but BMI class is different.

Conversely, multiple regression model has shown the negative influence of the type of insert on the survival of the prosthesis; the mobile bearing being worse than the fixed one, both when the endpoint was failure for any reason, and when the endpoint was sepsis. The decision to include the type of mobility of the insert in the analysis was prompted by evidence already observed both in the RIPO data and in the data of the Australian register that shows how the mobile insert is worse than the fixed one [17]. Indeed we confirmed that the rate-ratio (RR) was 1.88 when the end point was loosening for any reason, which increased to 2.28 when the end-point was septic loosening. The interpretation of this finding is not simple. The reason may be the smaller size of the polyethylene particles that are generated by mobile-bearing knees owing to the more conformed articular surface and additional undersurface wear. [23],

The same analysis showed that the survival of the prosthesis was positively influenced by the increase in the patient's age at surgery. In fact, an increase by only one year of age reduces the risk of loosening, possibly due to the reduction in the patient's motor activity. [14]

A possible bias in these results might derive from the observation that obese patients are less likely to be offered a revision. On the contrary we verified that surgeons, in the region where the study was conducted, do not refuse revision surgery even to obese patients. Only very severe co-morbidities, and not obesity itself, can prompt anesthetists to contraindicate anesthesia.

A comparison with the data of the literature can be made only with series collected in single centres. Large series of registers in fact are lacking, because BMI is not recorded by the registers that historically deal with the knee [14-17], or it is recorded but has not yet been elaborated, as in England, where the follow-up is too short.

On the whole, the panorama of experiences on this matter is variegated because the end-points, the classification of obesity, and the follow-ups are different and probably because the series rarely exceed a hundred patients. [24]

Taking the revision of the prosthesis as the end-point, our results agree with authors that have published mid-term studies, such as Amin, who compared 125 obese with 158 non-obese patients and concluded that simple obesity (BMI > 30 but < 40) did not influence the clinical outcome or revision rate 5 years after TKA [25], and Spicer, who studied 285 osteoarthritic obese patients at a mean follow-up of 6 years and observed that survivorship of the prostheses was similar to that of non-obese patients [26].

Foran [27] studied 68 obese and 68 non-obese patients, Kaplan Meier survivorship analysis revealed that obese and non-obese patients behave in a similar way up to 60–80 months from surgery (albeit with a better quality of life in non obese patients), and then a decrease in survival rate becomes apparent for the obese group.

Our results also agree with longer-term follow-up studies, up to ten years, where obese and non-obese patients have a similar pattern [28,29] and where TKA resulted in improved mobility, thus enhancing the chance of success of subsequent weight loss therapy [30].

Again with a long-term follow-up, other authors have found results that differ from ours despite using the same statistical method. Among them Foran who followed a smaller cohort of 27 non-obese and 27 obese patients with a cementless prosthesis for 15 years and found a trend not statistically significant for obesity to influence the rate of aseptic loosening, but a higher revision rate of poly liners in non-obese patients, probably due to a higher rate of activity [31]. Vazquez-Vela Johnson, followed 145 patients for more than 10 years and observed that age, gender and BMI all make a difference to the survival rate of osteoarthritic patients; male gender, obesity and age below 60 years are risk factors for prosthesis survival [32].

Concerning complications, Miric, found that heavier patients were more likely to experience a complication (38% versus 25%) and multiple complications (9.3% versus 6.2%) compared with lighter patients [33].

A longer follow-up might reveal phenomena that cannot be seen yet, despite the large size of the sample we have studied.

Another question is patients with morbid obesity (BMI > 40). Different conclusions have been reached also with regards to these patients, based on extremely small series. Krushell 2007 found that 39 patients with a BMI > 40 had higher rate of revision but did not reach statistical significance [34].

Conversely, Amin compared 41 obese with 41 morbidly obese patients for a mean of 38 months after knee replacement and found that the second group suffered a higher rate of complications and peri-implant radiolucency [35]. Winiarsky studied 40 morbidly obese patients with a minimum follow-up of 2 years and found more perioperative complications in these patients. [36]

Altogether the three Authors studied 120 patients, a very low number, as is the series reported in our study, which includes 172 morbidly obese patients. The shortage of patients in this group is related not only to the extreme condition they were in, but also the difficulty to find both orthopedic surgeons and anesthetists willing to treat these "risk" subjects.

None of the cited authors performed multivariate analysis, except for Winiarsky [36]. The data we have presented are certainly limited by the chosen end-point that is surgical revision of at least one component. Nothing is known about the quality of life and radiological aspects of the patients, another limit of the study is the short follow-up.

In fact the minimum follow-up of 18 months and maximum of 60 does not reveal the late phenomena of polyethylene wear, which, however, at least in this short follow-up, does not seem to penalize obese patients. Undoubtedly, the time of observation is limited in comparison with the life expectancy of the prosthesis; therefore it is not possible to extend the conclusions to the real duration of the prosthesis itself.

Further limits of the study are the lack of knowledge of the level of physical activity of the patients and all the other elements of clinical follow-up, such as degree of pain relief, weight change, smoking habits, besides the possible use of surgical navigator. Obese patients might practise a lower level of activity in comparison with normal weight patients which limits the wear of the polyethylene, thus compensating for the greater stress due to body weight, and patients that were obese during the operation might have lost some body weight thanks to the restoration of function of the replaced joint.

In our opinion, these limits are balanced by the great strength of a multicentric series of over 9700 arthroplasties performed with prostheses of different types, all totally cemented to treat a homogeneous series of osteoarthritic patients. Furthermore, our large series has allowed us to analyze the data with a multivariate statistical method, thereby testing the weight of the risk factors.

## Conclusion

The link between excessive body weight and health is an important concern for public health policy, because obesity is a well-known risk factor for many costly diseases such as cardiovascular disease, diabetes and some types of cancer.

Based on our evidence, however, it does not appear justified to give low priority to obese sujects for TKA which would, in virtue of restored ability to move, lead to weight loss, which undoubtedly, should be the primary objective.

In conclusion, according to the Register data and considering the limits that this type of data can have, at present it can be said that up to 5 years after surgery obese subjects do not run a higher risk of totally cemented knee prosthesis revision. These patients do not carry a greater risk of perioperative complications either. The conclusion also applies to subjects affected by morbid obesity, albeit with caution related to the small sample examined.

## Competing interests

The authors declare that they have no competing interests.

## Authors' contributions

BB and SC (statisticians) analysed the data, SS (biologist) coordinated data collection and, together with MV prepared the manuscript, MV (Engineer) gave his advice from a biomechanical point of view, AT (orthopedic surgeon) conceived the study and gave his clinical advice, RDP (public health medical doctor) collaborated to data collection and gave his advice on public health issues.

## Pre-publication history

The pre-publication history for this paper can be accessed here:


